# Risk exploration and prediction model construction for linezolid-resistant *Enterococcus faecalis* based on big data in a province in southern China

**DOI:** 10.1007/s10096-023-04717-3

**Published:** 2023-11-30

**Authors:** Peijun Liu, Bangwei Zeng, Xiaoyan Wu, Feng Zheng, Yangmei Zhang, Xiaohua Liao

**Affiliations:** 1 Administration Department of Nosocomial Infection, Fujian Medical University Union Hospital, Fuzhou, 350001 Fujian Province China; 2 Information Department, Fujian Medical University Union Hospital, Fuzhou, 350001 Fujian Province China

**Keywords:** *Enterococcus faecalis*, Linezolid resistance, Artificial neural network, Logistic regression, Risk factor

## Abstract

**Background:**

*Enterococcus faecalis* is a common cause of healthcare-associated infections. Its resistance to linezolid, the antibiotic of last resort for vancomycin-resistant enterococci, has become a growing threat in healthcare settings.

**Methods:**

We analyzed the data of *E. faecalis* isolates from 26 medical institutions between 2018 and 2020 and performed univariate and multivariate logistic regression analyses to determine the independent predictors for linezolid-resistant *E. faecalis* (LREFs). Then, we used the artificial neural network (ANN) and logistic regression (LR) to build a prediction model for linezolid resistance and performed a performance evaluation and comparison.

**Results:**

Of 12,089 *E. faecalis* strains, 755 (6.25%) were resistant to linezolid. Among vancomycin-resistant *E. faecalis*, the linezolid-resistant rate was 24.44%, higher than that of vancomycin-susceptible *E. faecalis* (*p* < 0.0001). Univariate and multivariate regression analyses showed that gender, age, specimen type, length of stay before culture, season, region, GDP (gross domestic product), number of beds, and hospital level were predictors of linezolid resistance. Both the ANN and LR models constructed in the study performed well in predicting linezolid resistance in *E. faecalis*, with AUCs of 0.754 and 0.741 in the validation set, respectively. However, synthetic minority oversampling technique (SMOTE) did not improve the prediction ability of the models.

**Conclusion:**

*E. faecalis* linezolid-resistant rates varied by specimen site, geographic region, GDP level, facility level, and the number of beds. At the same time, community-acquired *E. faecalis* with linezolid resistance should be monitored closely. We can use the prediction model to guide clinical medication and take timely prevention and control measures.

**Supplementary information:**

The online version contains supplementary material available at 10.1007/s10096-023-04717-3.

## Introduction

*E. faecalis* is a Gram-positive, facultatively anaerobic, catalase-negative bacteria that normally lives in the intestines of healthy humans [1] and also widely distributed in nature. However, it can survive under harsh conditions [2] due to its resilient and versatile features, especially its intrinsic resistance to various antimicrobials and acquisition of resistance traits [3], which may cause serious infections in hospitals and difficult to treat. At 2020, it had become the third most common gram-positive bacteria and the fifth most common pathogen in healthcare-associated infections (HAI) worldwide from CHINET surveillance [4, 5].

Linezolid, as the last-line drug for vancomycin-resistant *E. faecalis*, has been frequently used in the clinical treatment of various serious infections such as pneumonia, meningitis, or skin infections, and also used for treating difficult infections and multi-drug resistant tuberculosis [6, 7]. Although the incidence of linezolid-resistant *E. faecalis* in humans is still very low, the emergence of linezolid-transferable resistance genes *cfr*, *cfr(B)*, *cfr(C)*, *poxtA*, and *optrA* in recent years [8–11] has raised concerns about the impact of *E. faecalis* on linezolid drug resistance. Linezolid resistance can also be caused by mutations in the 23S rRNA gene, and all of the above genes are increasingly observed in food-producing animals, food of animal origin, and humans[12]. Some studies have reported high detection of linezolid-insensitive *E. faecalis*, even be as high as 18% in food-producing animals [13, 14]. Due to the abuse of antimicrobial drugs and poor prevention and control, the resistance rate of *E. faecalis* to linezolid may gradually increase. Therefore, we need to monitor and predict the epidemiology of linezolid- resistant *E. faecalis*. This study will build a predictive model for linezolid resistance, which can guide clinicians on how to use antibiotics rationally and empirically and decision makers on how to formulate prevention and control measures timely and effective.

## Methods

We used big data from the Hospital Infection Prevention and Control Center System (NICC), a real-time monitoring system that receives healthcare-association infection data from secondary and above medical institutions across the province, to conduct a retrospective observational study of *E. faecalis*. For avoiding systematic bias due to changes in hospital participation over time, we included data from 26 medical institutions which uploading relatively complete data for more than 36 months from 2018 to 2021.

### Selection of *E. faecalis* isolates

We extracted relevant data on *E. faecalis* reported by 26 medical institutions above from the system from 2018 to 2021. We selected only the first *E. faecalis* isolate from each inpatient and excluded duplicate isolates. We also excluded isolates that did not have linezolid susceptibility testing or had missing information on the factors. We defined linezolid resistant *E. faecalis* (LREfs) as those that were reported as linezolid-intermediate or linezolid-resistant by the medical institutions. We defined linezolid-susceptible *E. faecalis* (LSEfs) as those that were reported as linezolid-sensitive. Sensitivity judgments for other antibiotics also follow the above principles. The Clinical and Laboratory Standards Institute (CLSI) M100 standard is followed by the microbiological labs in the hospitals where we collected our data.

### Factors

We collected possible risk factors and categorized them. The age of patients was divided into four age groups (0–18, 19–45, 46–65, and 66-year-old). Patients were divided into female and male. The sources of isolates are divided into north, west, south, and east regions and are divided into two categories: 0–P50 and P51 or above according to the GDP of the source area. Isolate acquisition time is divided into spring, summer, autumn, and winter, according to the season, and divided into 5 categories (0–2, 3–7, 8–14, 15–28, and 29 days) according to the number of days of hospitalization when the pathogen was detected. The source of the isolate is divided into respiratory, urinary, cerebrospinal fluid, secretion, blood, drain fluid, and others. The hospital is divided into secondary-level hospital and tertiary-level hospital according to the type of hospital and divided into five categories according to the number of beds (0–500, 501–1000, 1001–1500, 1501–2000, and 2001 beds). The source department of isolates is divided into the intensive care unit and non-intensive care unit.

### Statistical analysis

We used SAS software (version 9.4, SAS Institute, Cary, NC, USA) or R software (version 4.2.1, R Foundation, Vienna, Austria) for statistical analyses. The primary outcome was the proportion of *E. faecalis* isolates that were not susceptible to linezolid (%). We reported continuous variables as means or medians, and categorical variables as frequencies or proportions. We used the Chi-squared test to compare the proportions of LREfs by different variables.

### SMOTE

Synthetic minority oversampling technique (SMOTE) is a common oversampling method that creates synthetic samples based on the features of the minority class instances and their nearest neighbors. SMOTE increases the number of minority class without affecting the number of majority class [15]. In this study, we used SMOTE to balance the training set so that the ratio of positive samples (LSEfs) to negative samples (LREfs) was close to 1:1.

### Predictive model construction

We randomly split the data set into a training set (70%) and an independent validation set (30%). We used univariate and multivariate logistic regression analyses for variable screening only in the training set. Factors that were statistically significant (a *p*-value less than 0.05 was considered significant) in the univariate logistic regression analysis were input variables in the artificial neural network (ANN) and multivariate analyses. Then, we used stepwise logistic regression for multivariate analysis to determine independent predictors. Both the ANN and logistic regression (LR) models were constructed on the same training set (with or without SMOTE) and tested on the same validation set.

We used RSNNS packages for R for ANN algorithms and the results of the multivariate analysis to build up the LR model. The dependent variable was LREfs rate. For internal validation, we used bootstrapping method. We used Youden index to determine accuracy, sensitivity, and specificity from the optimal threshold. And we measured accuracy, sensitivity, specificity, receiver operating characteristic (ROC) curve, area under the ROC curve (AUROC), calibration curves, leave-one-out cross validation, and decision curve analysis (DCA) on validation set to evaluate model performance [16]. The difference in AUROC values between the two models was compared with the method described by DeLong et al.

## Results

We collected 364,886 isolates from clinical specimens of inpatients in 26 medical institutions from 2018 to 2021. Of these, 13,556(3.72%) were *E. faecalis*. 12,089 (89.18%) isolates of *E. faecalis* were tested to linezolid. A total of 755 isolates of LREfs were detected, with a resistant rate of 6.25%.

### Drug susceptibility of *E. faecalis*

**Fig. 1:**
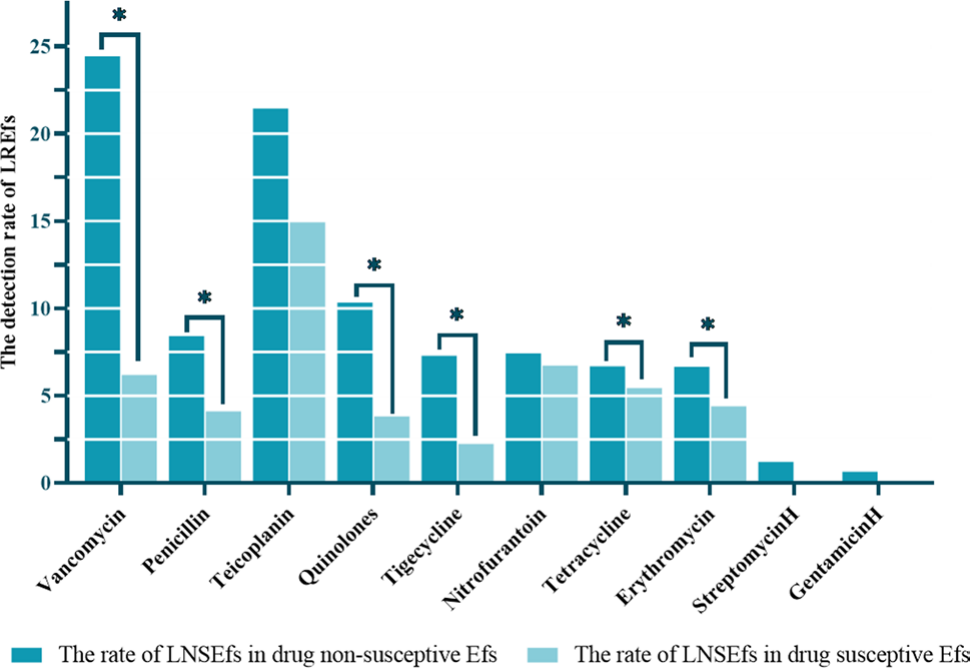
Resistance of different antibiotic-resistant *E. faecalis* to linezolid. LREFs: linezolid-resistant *E. faecalis*. *The difference is statistically significant

Among vancomycin-resistant *E. faecalis*, the linezolid resistance rate was 24.44%, which was higher than that of vancomycin-susceptible *E. faecalis* (*p* < 0.001). In addition, among *E. faecalis* resistant to penicillin, quinolones, tigecycline, tetracycline, or erythromycin, the rate of resistance to linezolid was also higher than that of sensitive strains, with statistical significance (*p* < 0.05) (shown in Fig. 1).

### Correlative analysis of clinical data of LREfs

#### Basic characteristics of predictive factors related to LREfs

The 755 specimens of LREfs were mainly from people older than 46 (median age 59), with a male/female ratio of 1.23. There is no significant difference in distribution. Most of these specimens came from hospitals in the eastern region, tertiary hospitals, or hospitals with beds between 1501 and 2000. The specimens of LREfs obtained from non-ICU inpatients were the most, up to 88.48%. The number of samples of LREfs detected within 2 days after admission accounted for 49.27%. The number of specimens in spring, summer, and autumn was not much different, and the number of LREfs detected in winter was relatively small. The specimens were mainly from the respiratory system (49.27%), followed by urine (44.37%) and secretion (28.48%). The baseline characteristics of *E. faecalis* and LREfs are shown in supplemental Table A1.

#### Univariate and multivariate analyses of predictive factors for LREfs

**Fig. 2:**
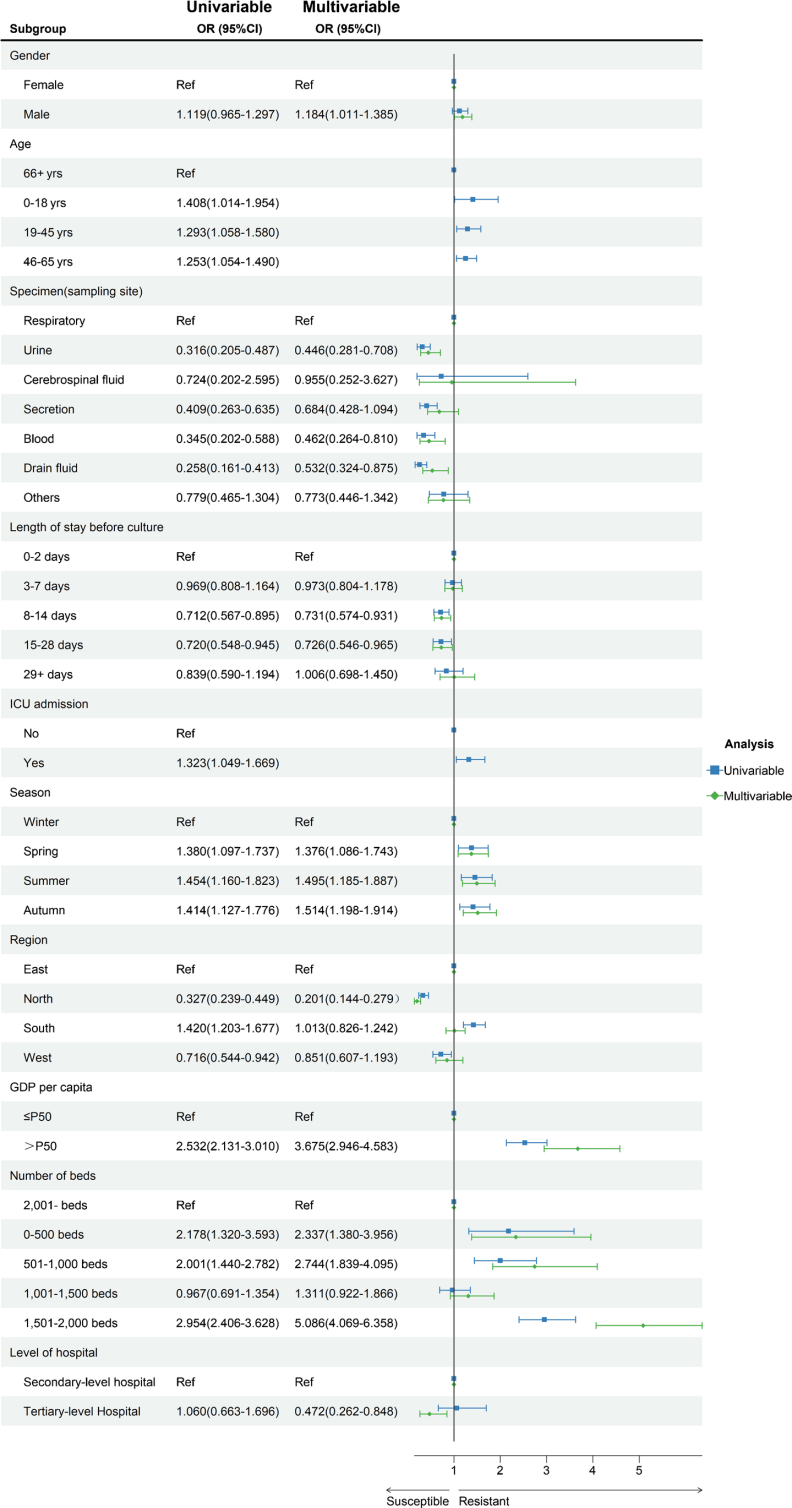
Univariable and multivariable analyses of factors associated with LREfs

Male and GDP > P50 were independent predictors (risk factors) of *E. faecalis* resistant to linezolid, while tertiary hospitals and winter were independent protective variables, according to univariate and multivariate regression analysis. Additionally, compared to *E. faecalis* isolates from respiratory specimens, *E. faecalis* isolates from drainage fluid, blood, and urine were less likely to be linezolid resistant. In comparison to hospitals with more than 2000 beds, those with fewer than 500 beds, 501–1000 beds, and 1501–2000 beds were more likely to find LREf isolates. The rate of LREf isolates in the northern region was lower than in the eastern region (showed in Fig. 2).

#### Changes in detection rate of LREfs with days of hospitalization in different GDP regions

**Fig. 3:**
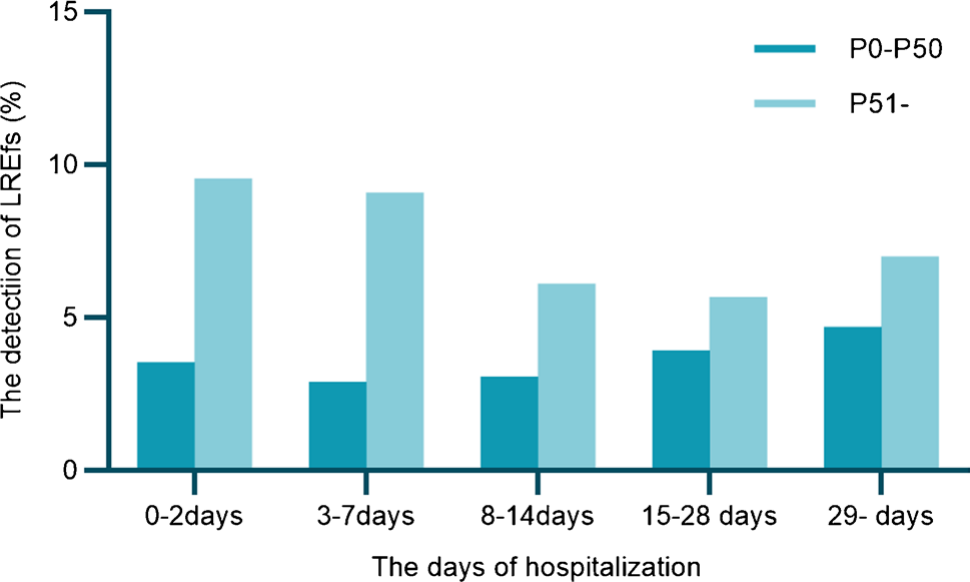
Changes in detection rate of LREfs with days of hospitalization in different GDP regions. LREFs: linezolid-resistant *E. faecalis*; P0–P50: means GDP ranking in 0–50%; P51-: means GDP ranking above 50%

Within 2 days of hospitalization, the resistant rate of *E. faecalis* from low-GDP regions was 3.54%, which was comparable to the rate observed after 2 days of hospitalization (3.36%). However, the rate of LREfs within 2 days in high-GDP areas was statistically higher than that after 2 days (9.56% vs. 7.35%; *p* < 0.001) (showed in Fig. 3).

### A predictive model for LREfs

**Fig. 4:**
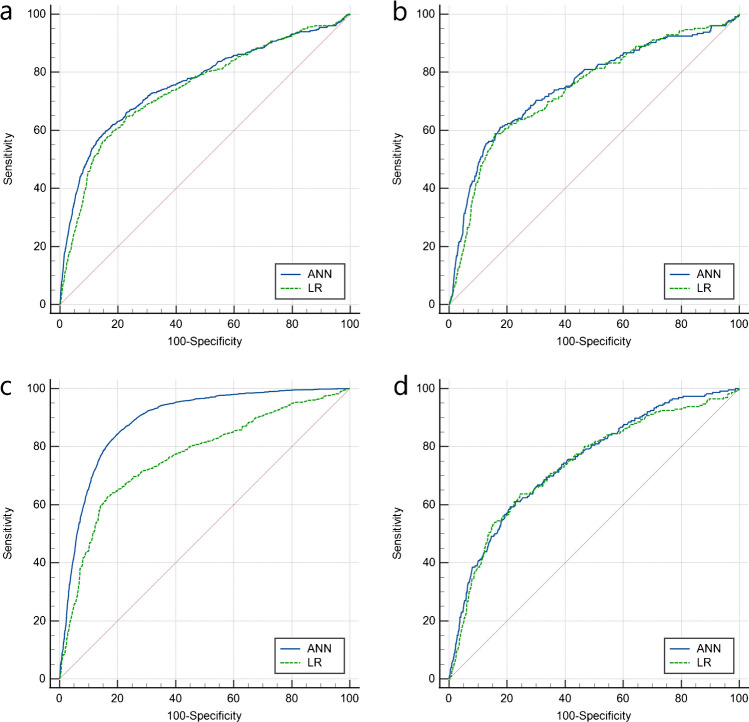
Receiver-operating characteristic curves (ROC) for the ANN and LR predictive models. LR: logistic regression; ANN: artificial neural network; SMOTE: synthetic minority oversampling technique; LREFs: linezolid-resistant *E. faecalis*. **A** ROC of LREFs predictive model constructed by artificial neural network and logistic regression models in training set. Differences between ANN and LR models were statistically significant (*p* < 0.001). **B** ROC of LREFs predictive model constructed by artificial neural network and logistic regression models in validation set. Differences between ANN and LR models were not statistically significant (*p* = 0.1505). **C** ROC of LREFs predictive model constructed by artificial neural network and logistic regression models in training set after the training set was processed using the SMOTE. Differences between ANN and LR models were statistically significant (*p* < 0.001). **D** ROC of LREF predictive model constructed by artificial neural network and logistic regression models in validation set after the training set was processed using the SMOTE. Differences between ANN and LR models were not statistically significant (*p* = 0.4443)

**Fig. 5:**
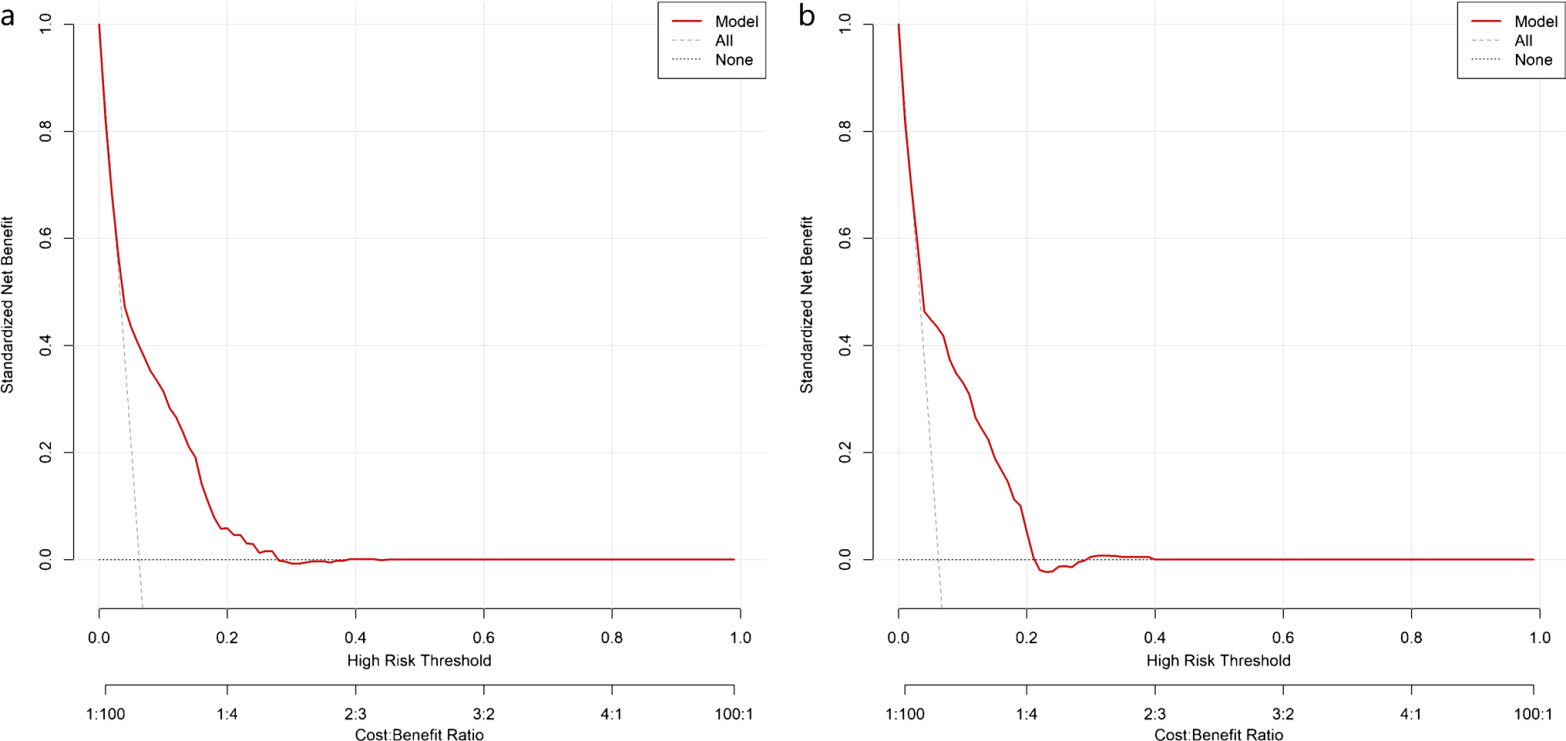
Decision curve analysis (DCA) curve of the LR model for predicting LREFs. **A** The DCA curve of the LR model for predicting LREFs in training set. **B** The DCA curve of the LR model for predicting LREFs in validation set. The *y*-axis represents the net income, the *x*-axis represents the threshold probability, and the red line represents the model. The gray line indicates that no patients are assumed to have LREFs, the black line indicates that all patient are assumed to have LREFs, and the red line indicates the results of the LREF prediction model. In validation set, the final DCA shows that if the threshold probability is between 0 and 21%, the strategy based on LR model to predict the rate of LREFs in this study produces better net benefits than the “all LREFs” and “no LREFs” modes. In this range, the prediction effect of the model is the best

In the ANN prediction model, an input layer consisting of 23 neurons (including the 23 variables found in the univariable regression analysis) was parsed. The number of neurons in the hidden layer was determined to be 18 (showed in Fig. 2). And the LR prediction model was constructed by utilizing the independent predictors discovered in a multivariable investigation. In the training set, the AUC of ANN was greater than that of LR, and the difference was statistically significant (0.763 vs. 0.745, *p* < 0.001). But the predictive performance of the two models was comparable when predicted on the validation set. Additionally, after the training set was processed using the SMOTE, the predictive performance of the ANN and LR prediction models in the training set both not improved but decreased in the validation set (showed in Fig. 4). The calibration plot shows good predictive accuracy between the actual probability and predicted probability (showed in supplementary Figure A2). For the LR model, we use leave-one-out cross-validation to verify the generalization ability, and get AUC = 0.7311, additionally we evaluated the model’ clinical utility with DCA method, and the results showed that when the probability of LREFs is between 0 and 21%, the prediction model has good clinical utility (showed in Fig. 5). And the prediction performance of ANN and LR based on the optimal feature subset is compared through the sensitivity, specificity, accuracy, precision, F1 value, and AUC that are shown in Table 1.

**Table 1:** Performance of the ANN and LR prediction model on 755 LREfs in a north province of China during 2018–2021

		Without SMOTE			With SMOTE		
		ANN (95% CI)	LR (95% CI)	LR vs. ANN	ANN (95% CI)	LR (95% CI)	LR vs. ANN
Train	Sensitivity	59.74%	64.65%		84.19%	62.76%	
	Specificity	84.38%	77.14%		80.29%	83.46%	
	Accuracy	82.84%	76.36%		50.00%	50.00%	
	Precision	20.32%	15.86%		81.03%	79.14%	
	F1-score	30.33%	25.47%		63.66%	70.01%	
	Youden index *J*	0.4412	0.4179		0.6448	0.4622	
	AUROC	0.763 (0.754–0.772)	0.745 (0.735–0.754)	*Z* = 3.334, *p* = 0.0009	0.890 (0.883–0.896)	0.764 (0.755–0.773)	*Z* = 28.415, *p* < 0.0001
Validation	Sensitivity	61.06%	58.85%		59.29%	63.72%	
	Specificity	82.38%	84.21%		78.94%	75.44%	
	Accuracy	81.05%	87.87%		77.72%	74.71%	
	Precision	18.72%	27.71%		15.76%	14.71%	
	F1-score	28.66%	37.68%		24.91%	23.90%	
	Youden index *J*	0.4344	0.4306		0.3823	0.3916	
	AUROC	0.754 (0.739–0.768)	0.741 (0.726–0.755)	*Z* = 1.438, *p* = 0.1505	0.746 (0.732–0.761)	0.736 (0.721–0.750)	*Z* = 0.765, *p* = 0.4443

## Discussion

In the analysis of the drug susceptibility results of *E. faecalis*, bacteria that were resistant to vancomycin, penicillin, quinolones, tigecycline, tetracycline, and erythromycin seem to be more likely to be resistant to linezolid. Several studies have demonstrated that certain resistance genes associated with linezolid resistance, such as *cfr* and *optrA*, can confer resistance to multiple antibacterial agents simultaneously. Furthermore, the presence of IS256-like sequences may obscure additional resistance genes. Therefore, further research is necessary to elucidate the relationship between these antibiotic resistances [17, 18]. The probability of linezolid insensitivity in patients with the aforementioned antimicrobial drug resistance, however, may be increased by risk factors such immunocompromised individuals, the administration of numerous antibiotics, a prolonged hospital stay, and ICU admission[19]. On the other hand, they are increasingly resistant not only to antibiotics but also disinfectants which is complicating infection control recommendations [20, 21]. We must also mention that none of the *E. faecalis* from the 26 medical institutions in this study were tested for daptomycin susceptibility, which limits its use for *E. faecalis* infections and increases the options for linezolid and resistance to linezolid [22–25].

Our study also found that LREfs in high-income areas are higher than those in low-income areas, which is contrary to other studies [26, 27]. This may be due to more antimicrobial use and environmental contamination selecting for bacterial resistance in high-income areas. Additionally, LREfs from the community was also high in this study, specifically in high-income areas, and LREf intestinal tract colonization as a reservoir of LREfs acquired through the community may be asymptomatic but may occur infection in hospitals [28]. Sometimes LREfs carrying silent *cfr* and/or *optrA* from the community may be hard to detect in clinical setting [29], but may cause nosocomial outbreaks of LREfs infections [30, 31], endangering patients’ lives.

According to our multifactor analysis, we included eight variables to construct predictive models for LREfs using ANN and LR. The ANN model performed better on the training set, but both models performed similarly on the validation set with good predictive performance and robustness. The DCA curve of the LR model showed a better net benefit when the threshold probability was between 0 and 21%. We also used SMOTE technology to balance the data and improve the performance of the model in the validation set. After SMOTE processing, various performance indicators of the ANN model on the training set improved significantly. However, performance indicators on the validation set decreased instead. The SMOTE technique may cause overfitting and fail to generalize to new and unseen data. The prediction performance of the LR model following SMOTE treatment also did not improve.

**Fig. 6:**
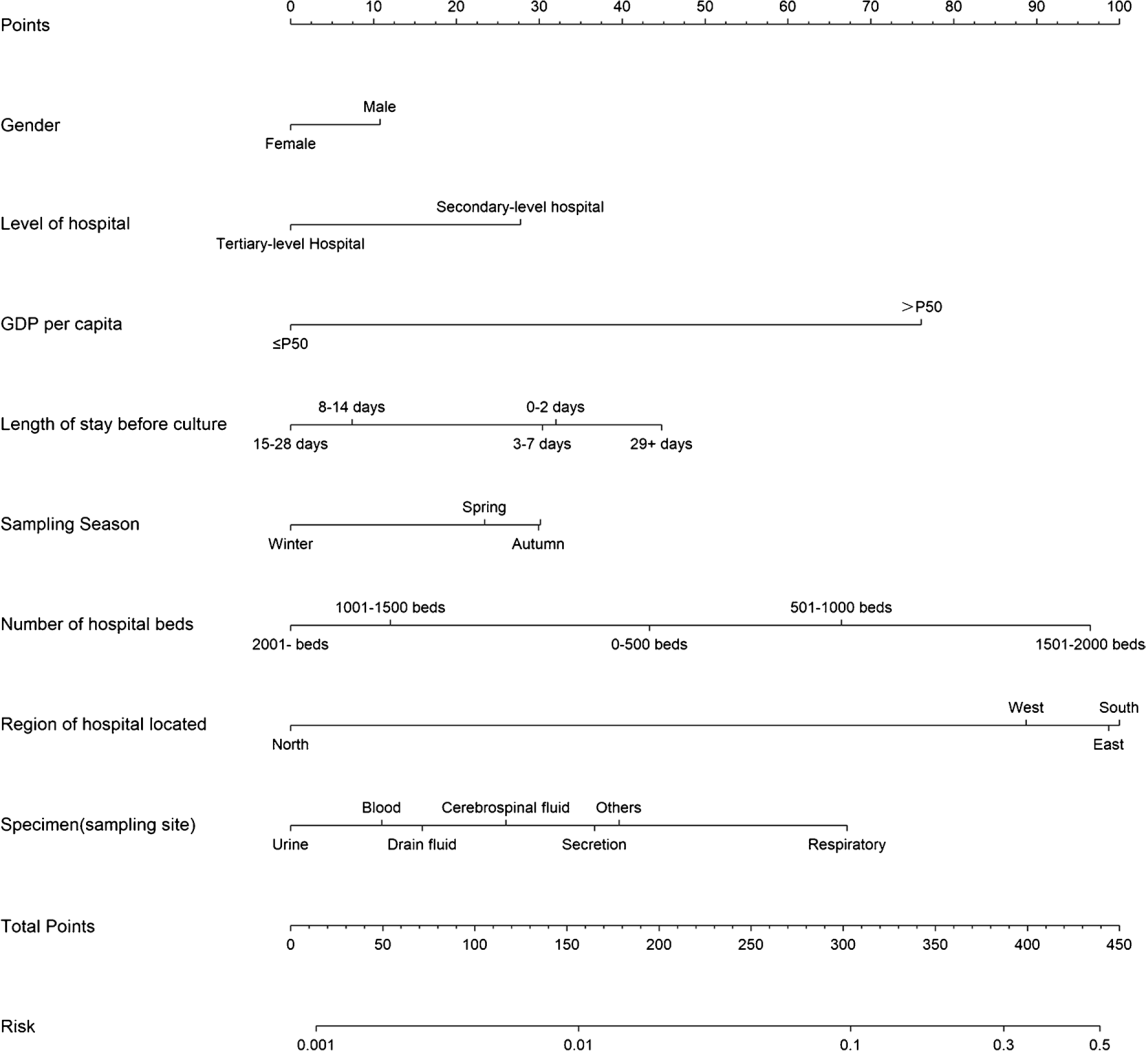
A nomogram for predicting the proportion of LREFs

We believe that our model can help clinical doctors to choose drugs reasonably after being applied to clinical practice and take effective prevention and control measures in a timely manner for high-risk populations who are insensitive to metronidazole, thereby reducing the burden of metronidazole resistance on patients and medical treatment. We also provided a nomogram diagram (showed in Fig. 6) for real-world use. This model’s advantages include ease of use and affordability, which can promote its use and popularity.

This study has some limitations: firstly, we did not have more individualized data for patients from each medical institution, such as tracheal intubation, central venous catheter, urine catheter, or surgery. Therefore, we did not include these factors in the study. Secondly, since our data comes from a big data platform, this database follows the principle of minimizing data and only collects the final results of the MIC values of pathogenic bacteria judged by various medical institutions’ microbiology laboratories according to CLSI standards. Therefore, we regret that we cannot obtain specific MIC values for more similar analysis. Thirdly, simple SMOTE technology cannot improve the predictive performance of the model, and more processing techniques need to be tried.

### Supplementary information

Below is the link to the electronic supplementary material.

Supplementary file1 (TIF 334 KB) Supplementary Figure A2 The calibration plot of the logistic regression and ANN prediction models. a: The calibration plot of the Logistic regression and ANN prediction models without SMOTE. b: The calibration plot of the Logistic regression and ANN prediction models with SMOTE.

‘Supplementary file2 (DOCX 21 KB) Supplementary Table A1 Basic characteristics of predictive factors related to *E. faecalis.*

## Data Availability

The datasets generated during and/or analyzed during the current study are not publicly available but are available from the corresponding author on reasonable request.
