# Supplementary figures and images for: Risk exploration and prediction model construction for linezolid-resistant Enterococcus faecalis based on big data in a province in southern China
Source: Eur J Clin Microbiol Infect Dis. 2023 Nov 30;43(2):259–68. doi: 10.1007/s10096-023-04717-3 (PMC10821975; doi:10.1007/s10096-023-04717-3)

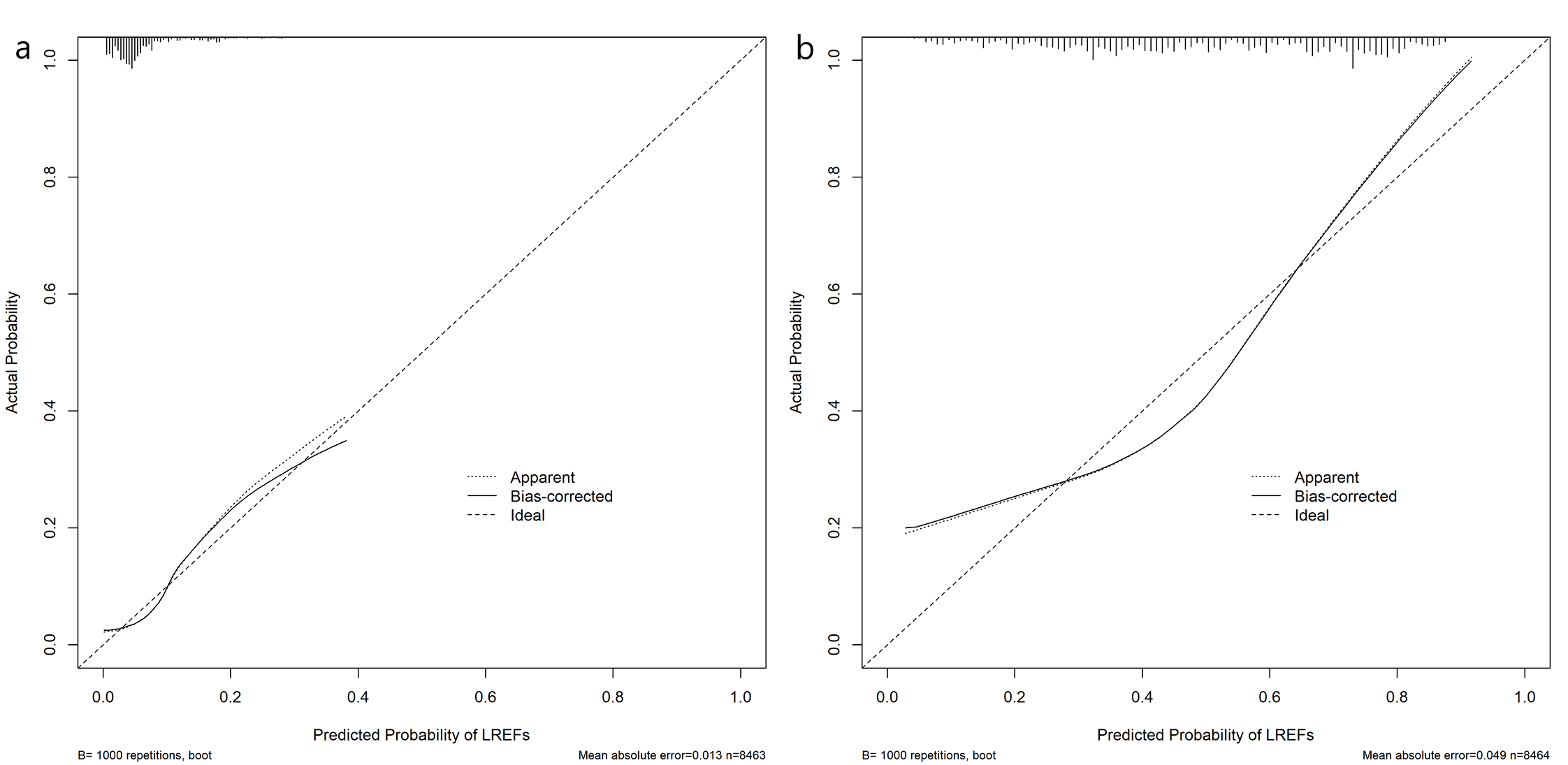

Supplement: Supplementary file 1 — Supplementary file1 (TIF 334 KB) Supplementary Figure A2 The calibration plot of the logistic regression and ANN prediction models. a: The calibration plot of the Logistic regression and ANN prediction models without SMOTE. b: The calibration plot of the Logistic regression and ANN prediction models with SMOTE. [file 10096_2023_4717_MOESM1_ESM.tif]
