# Supplementary material for: Risk exploration and prediction model construction for linezolid-resistant Enterococcus faecalis based on big data in a province in southern China
Source: Eur J Clin Microbiol Infect Dis. 2023 Nov 30;43(2):259–68. doi: 10.1007/s10096-023-04717-3 (PMC10821975; doi:10.1007/s10096-023-04717-3)
Supplement: Supplementary file 2 — ‘Supplementary file2 (DOCX 21 KB) Supplementary Table A1 Basic characteristics of predictive factors related to E. faecalis. [file 10096_2023_4717_MOESM2_ESM.docx]

Supplementary Table A1 Basic characteristics of predictive factors related to *E. faecalis*

|  | *E. faecalis* | | LNSEfs | |
| --- | --- | --- | --- | --- |
|  | n  (N total=12,089) | % | n  (N total=755) | % |
| Gender |  |  |  |  |
| male（n,%） | 6,345 | 52.49 | 416 | 55.10 |
| femal（n,%） | 5,744 | 47.51 | 339 | 44.90 |
| Sex ratio (m/f) | 1.11 |  | 1.23 |  |
| Age |  |  |  |  |
| 0-18 yrs（n,%） | 604 | 5.00 | 45 | 5.96 |
| 19-45 yrs（n,%） | 2,396 | 19.82 | 165 | 21.85 |
| 46-65 yrs（n,%） | 4,172 | 34.51 | 279 | 36.95 |
| 66- yrs（n,%） | 4,917 | 40.67 | 266 | 35.23 |
| median Age | 61 |  |  |  |
| Specimen(sampling site) |  |  |  |  |
| Respiratory（n,%） | 164 | 1.36 | 26 | 3.44 |
| Urine（n,%） | 5,961 | 49.31 | 335 | 44.37 |
| Cerebrospinal fluid（n,%） | 25 | 0.21 | 3 | 0.40 |
| Secretion（n,%） | 3,008 | 24.88 | 215 | 28.48 |
| Others（n,%） | 383 | 3.17 | 49 | 6.49 |
| Blood（n,%） | 607 | 5.02 | 37 | 4.90 |
| Drain fluid（n,%） | 1,941 | 16.06 | 90 | 11.92 |
| Length of stay before culture | | | | |
| 0-2days（n,%） | 5,438 | 44.98 | 372 | 49.27 |
| 3-7days（n,%） | 2,784 | 23.03 | 185 | 24.50 |
| 8-14days（n,%） | 1,972 | 16.31 | 98 | 12.98 |
| 15-28 days（n,%） | 1,275 | 10.55 | 64 | 8.48 |
| 29- days（n,%） | 620 | 5.13 | 36 | 4.77 |
| ICU admission |  |  |  |  |
| No（n,%） | 10,986 | 90.88 | 668 | 88.48 |
| Yes（n,%） | 1,103 | 9.12 | 87 | 11.52 |
| Season |  |  |  |  |
| Spring（n,%） | 3,067 | 25.37 | 199 | 26.36 |
| Summer（n,%） | 3,230 | 26.72 | 220 | 29.14 |
| Autumn（n,%） | 3,179 | 26.30 | 211 | 27.95 |
| Winter（n,%） | 2,613 | 21.61 | 125 | 16.56 |
| Region |  |  |  |  |
| East（n,%） | 6,198 | 51.27 | 408 | 54.04 |
| North（n,%） | 1,951 | 16.14 | 44 | 5.83 |
| South（n,%） | 2,649 | 21.91 | 241 | 31.92 |
| West（n,%） | 1,291 | 10.68 | 62 | 8.21 |
| GDP per capita |  |  |  |  |
| P0-P50（n,%） | 5,106 | 42.24 | 176 | 23.31 |
| P51-（n,%） | 6,983 | 57.76 | 579 | 76.69 |
| Number of beds |  |  |  |  |
| 0-500 beds（n,%） | 280 | 2.32 | 19 | 2.52 |
| 501-1,000 beds（n,%） | 877 | 7.25 | 55 | 7.28 |
| 1,001-1,500 beds（n,%） | 1,597 | 13.21 | 50 | 6.62 |
| 1,501-2,000 beds（n,%） | 5,718 | 47.30 | 514 | 68.08 |
| 2,001- beds（n,%） | 3,617 | 29.92 | 117 | 15.50 |
| Level of hospital |  |  |  |  |
| Secondary-level hospital（n,%） | 321 | 2.66 | 19 | 2.52 |
| Tertiary-level Hospital（n,%） | 11,768 | 97.34 | 736 | 97.48 |
